# Disease-Free Survival of Patients with Stage II Stroma-Rich Colorectal Adenocarcinomas with Microsatellite Stability

**DOI:** 10.3390/ijms262411795

**Published:** 2025-12-06

**Authors:** Ángel Romo-Navarro, Juan Ruiz Martín, Irene García-Camacha Gutiérrez, Mariano Amo-Salas, María Recuero Pradillo, César Sánchez-Muñoz, Cristina María Murillo Lázaro, Esperanza Carabias López, Raquel Sánchez Simón, Carlos Quimbayo-Arcila, Yasmina Hernández Martín, María-Sonsoles Opazo Rodríguez, Yolanda Campos-Martín

**Affiliations:** 1 Department of Pathology, Complejo Hospitalario Universitario de Toledo, 45007 Toledo, Spain; 2 Department of Medicine, University of Alcalá, 28805 Madrid, Spain; 3 Department of Medicine, University of Castilla-La Mancha, 45004 Toledo, Spain; 4 Department of Mathematics, University of Castilla-La Mancha, 13071 Ciudad Real, Spain; 5 Hospital Nacional de Parapléjicos, 45071 Toledo, Spain; 6 Department of Inorganic Chemistry, Organic Chemistry and Biochemistry, Faculty of Environmental Sciences and Biochemistry, University of Castilla-La Mancha, 45004 Toledo, Spain

**Keywords:** colorectal cancer, stage II, stroma, mesenchymal

## Abstract

Up to 25% of stage II colorectal adenocarcinomas recur within the first five years after diagnosis. The assessment of the stromal percentage, recently incorporated into the TNM classification system, may represent a particularly relevant predictive factor for recurrence in cases with microsatellite stability. We evaluated disease-free survival (DFS) in an independent retrospective cohort, measured the stromal percentage across the entire invasive front of the tumor, and assessed the interobserver agreement of this measurement method. Among 131 cases, 16 (12.2%) showed a high stromal percentage and 115 (87.8%) a low one. A high stromal percentage was associated with high-grade budding (*p* = 0.006). The 5-year DFS was 57% for high-stroma cases versus 76% for low-stroma cases (*p* < 0.001). Lymphatic invasion (HR: 16.513; *p* < 0.001) and a high stromal percentage (HR: 4.366; *p* = 0.006) had a statistically significant correlation with DFS. Interobserver agreement for global stromal assessment was very good (kappa index = 0.870; *p* < 0.001). In conclusion, the stromal percentage may be a predictive factor for recurrence, particularly relevant in stage II microsatellite-stable colorectal adenocarcinomas. Global stromal assessment appears to be a simple and easily reproducible method.

## 1. Introduction

Colorectal cancer is one of the neoplasms with the highest incidence and mortality rates worldwide [1], and the number of cases is expected to increase in the coming years [2]. With current screening programs, up to 70% of cases are diagnosed at early stages without lymph node involvement [3]. Nevertheless, approximately 10–25% of stage II tumors recur within the first five years after diagnosis [4], showing poorer survival outcomes than some stage III cases [5]. In accordance with this, several high-risk factors for stage II disease have been defined, such as pT4 stage or fewer than 12 isolated lymph nodes, among others [6].

Recently, the European Society for Medical Oncology (ESMO) highlighted the importance of the association between a high stromal percentage (>50%) and worse disease-free survival (DFS) [7]. The Union for International Cancer Control (UICC) proposed the tumor–stroma ratio as an additional, nonessential prognostic factor in the 9th edition of its TNM tumor staging system [8].

A high stromal percentage defines a subset of colorectal cancers known as stroma-rich or mesenchymal tumors [9]. On a molecular level, these tumors are characterized by hyperactivation of the transforming growth factor beta (TGFβ) signaling pathway [10]. Under normal conditions, free TGFβ binds to the TGFβRII receptor, which dimerizes with TGFβRI—both membrane proteins with serine/threonine kinase activity. This activates the SMAD2/3/4 trimeric complex, which translocates to the nucleus to activate or inhibit various transcription-related genes, and is later degraded by ubiquitination [11]. In tumor-related hyperactivation, either due to excessive TGFβ or TGFβRII hyperactivation, alternative pathways, such as the RAS–MAPK or PI3K–AKT pathways, are triggered. These modulate processes involved in the epithelial–mesenchymal transition, affecting both epithelial and stromal cells [12].

At the epithelial level, cells in these tumors undergo changes in cytoskeletal proteins, replacing the membrane adhesion protein E-cadherin with others, such as N-cadherin or ZEB1, thereby acquiring a spindle-like morphology and increased motility [13]. Additionally, their secretion of metalloproteinases increases, facilitating the easier degradation of type IV collagen in the basement membrane and thereby enhancing metastatic potential [14]. These changes are related to the epithelial–mesenchymal transition and are reflected in the tumor budding biomarker, which can be measured at the tumor invasive front.

At the stromal level, latent fibroblasts become activated into cancer-associated fibroblasts (CAFs). CAFs acquire a contractile, proliferative, and profibrogenic phenotype, synthesizing large amounts of collagen that hinder the penetration of immune cells and drugs into the tumor core [15]. Moreover, CAFs interact with epithelial cells to form niches that facilitate more efficient metastatic migration [16].

Regarding immune cells, the recruitment of lymphocytes, monocytes, and polymorphonuclear cells is induced, but their activity becomes suppressed [17]. Th1 lymphocytes are depleted by fibroblast activation protein expressed by CAFs [18], and macrophages shift from the antitumor M1 to the immunosuppressive M2 phenotype [19].

Angiogenesis is also promoted through the induction of vascular endothelial growth factors (VEGFs) in endothelial cells. VEGF-A stimulates angiogenesis, while VEGF-C and VEGF-D promote lymphangiogenesis [14,15]. Additionally, pericyte recruitment is enhanced, stabilizing newly formed vessels and increasing the tumor’s metastatic potential [20].

All these epithelial and stromal alterations consume large amounts of energy, generating a hypoxic tumor microenvironment. This hypoxia leads to the acidification of cellular membranes, hindering the passage of chemotherapeutic agents, and induces neovascularization through the activation of HIF-1α [21,22].

Consequently, stroma-rich colorectal tumors display greater metastatic capacity and intrinsic chemoresistance to conventional treatments.

Stromal percentage assessment using hematoxylin–eosin (H&E) staining is a rapid and simple procedure, and its evaluation is generally conducted at the invasive tumor front [23]. Some researchers propose stromal assessment within a single field, while others recommend evaluation of the entire section [24].

Therefore, we hypothesize that determining the stromal percentage as a prognostic factor for recurrence in stage II tumors could provide highly relevant information, particularly in microsatellite-stable cases. Tumors with microsatellite instability account for 15% of all tumors, and develop through molecular pathways that induce a large number of mutations—which are sometimes deleterious to tumor survival—and respond effectively to treatments such as anti-PD-L1 and anti-CTLA-4 agents [25,26]. In contrast, microsatellite-stable tumors, which comprise 85% of the total, comprise a heterogeneous group with less successful targeted treatments and could benefit from additional biomarkers to help stratify recurrence risk in this majority group [24].

Through an independent retrospective cohort, our objectives were to (1) assess the association between stromal percentage and other clinicopathological variables of interest; (2) evaluate the prognostic role of stromal percentage in DFS; and (3) analyze interobserver agreement for both the single-field and global measurement methods.

## 2. Results

We defined a high stromal percentage as greater than 50% on H&E staining. Since West et al. first used a cutoff of 47% to distinguish between high and low stromal content [27], most studies have adopted the 50% threshold [28]. Some authors have proposed three categories (low, moderate, and high) [29], while artificial intelligence-based studies have identified an optimal cutoff value of 48.8% to discriminate between high and low stroma [30]. We used the 50% threshold because it is very close to the optimal value and is well established in the literature.

A total of 131 cases of stage II colorectal adenocarcinoma with microsatellite stability were diagnosed, with a median follow-up of 161 months.

**Figure 1:**
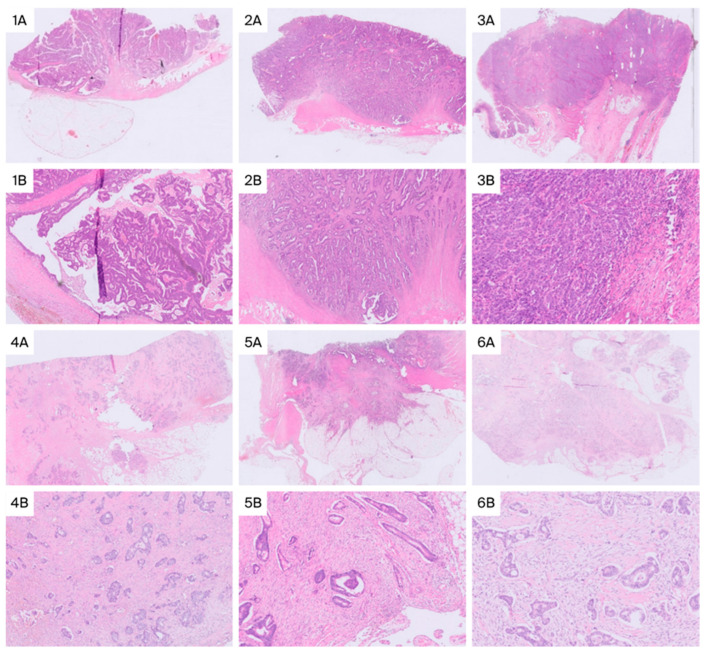
Examples of colorectal adenocarcinomas with low stromal percentage (≤50%) at low (**1A**,**2A**,**3A**) and high magnification (**1B**,**2B**,**3B**), and with high stromal percentage (>50%) at low (**4A**,**5A**,**6A**) and high magnification (**4B**,**5B**,**6B**).

Of these, 16 cases (12.2%) had a high stromal percentage and 115 (87.8%) had a low stromal percentage (Figure 1).

A high stromal percentage was significantly associated with high-grade tumor budding (*p* = 0.006), but not with any of the other clinicopathological variables (Table 1).

**Figure 2:**
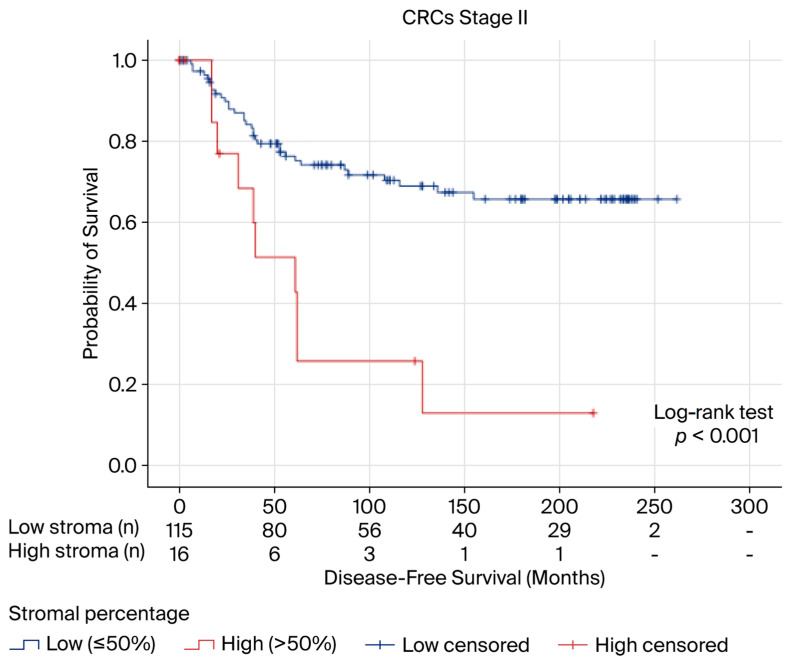
Disease-free survival curves for stage II microsatellite-stable colorectal adenocarcinomas (CRCs) according to stromal percentage.

The 5-, 10-, and 15-year DFS rates were 57%, 29%, and 17%, respectively, for high-stroma cases, and 76%, 69%, and 66%, respectively, for low-stroma cases. Median survival times were not calculated due to an insufficient number of recurrences in the low-stroma group. Kaplan–Meier survival curves showed significant separation between the two groups (*p* < 0.001) (Figure 2).

In the Cox regression analysis, only lymphatic invasion (HR = 16.513; 95% CI 3.506–77.769; *p* < 0.001) and a high stromal percentage (HR = 4.366; 95% CI 1.516–12.576; *p* = 0.006) were found to have a statistically significant impact on the risk of recurrence (Table 2).

Interobserver agreement for the global stromal measurement method was very good (kappa index = 0.870; *p* < 0.001). Agreement was moderate when the single-field selection method was used (kappa index = 0.525; *p* < 0.001).

## 3. Discussion

We investigated the role of the tumor stroma as a prognostic factor in colorectal adenocarcinoma, focusing on stage II tumors with microsatellite stability, based on H&E.

Other studies have explored stromal microRNAs in stage II tumors as an alternative to H&E assessment, reporting poorer outcomes associated with tumors with high expression of miR-21 and miR-556 [31,32].

Regarding associations with other clinicopathological characteristics, we found a statistically significant relationship only between a high stromal percentage and high-grade tumor budding (*p* = 0.006), a finding that has been repeatedly described in the literature [24,33]. Zengin et al. found this association particularly useful for predicting recurrence in stage I patients over 75 years of age, in whom adjuvant therapy is often limited by comorbidities [34]. Both phenomena are related to epithelial–mesenchymal transition, and interobserver agreement is higher for stromal assessment than for tumor budding evaluation [35].

Associations between high stromal percentage and lymphatic, vascular, and perineural invasion, as well as higher pT stages, have also been reported [36,37], although we did not observe these in our cohort. Other correlations, such as higher pN stage and peritoneal carcinomatosis, were exclusion criteria in this study [37].

In prognostic terms, we observed markedly worse DFS in stage II microsatellite-stable tumors with a high stromal percentage—57% at 5 years compared with 76% in those with low stromal percentage (*p* < 0.001). A poorer prognosis has been associated with a high stromal component across all stages [38,39,40,41], particularly in cases where lymph node metastases also showed a high stromal percentage [36]. Huijbers et al. found a 14% increase in high-risk patients when stromal assessment was included alongside other prognostic criteria [38].

In studies specifically addressing stage II disease, this adverse prognostic effect of high stromal content is consistently reported [33,42,43,44], with some emphasizing the relevance of the myxoid stromal subtype [45].

However, not all studies have reached the same conclusions. Dang et al. found no such association in 261 T1 patients [46], and Fekete et al. also failed to detect it in 74 stage II and III cases, attributing this to the small sample size [47]. Martin et al. reported the opposite association—an elevated epithelial component correlated with poorer outcomes (*p* = 0.0042)—leading to the definition of the Stroma AReactive Invasion Front Areas (SARIFA) concept [48,49]. SARIFA suggests that direct contact between epithelial cells and adipose tissue, once beyond the stroma, drives aggressive tumor behavior. Subsequent studies obtained similar results [50], suggesting that CAFs might also act as a barrier to tumor progression, leading to a proposed subclassification into tumor-promoting, tumor-restraining, and neutral CAFs [51]. This concept aligns with pancreatic cancer studies in mice, where stromal depletion accelerated rather than inhibited tumor progression [52,53,54]. Our study did not assess specific stromal subtypes or the presence of SARIFA. Future studies including scRNA-seq and multiplex IHC could help achieve a better characterization of pro-tumorigenic and anti-tumorigenic CAFs, enhancing the understanding of tumor heterogeneity and the mechanisms leading to poorer DFS.

Finally, stromal percentage has been measured both in a single field at the invasive front [23] and globally across the section [24]. One of the limitations of using the single- field method is that it could overestimate the stromal percentage due to tumor heterogeneity, classifying an entire tumor as stroma-rich based on a very small and unrepresentative area. We consider that a more holistic assessment better represents tumor heterogeneity and improves interobserver agreement. Using the single-field method, we obtained moderate agreement (kappa = 0.525), consistent with most reports (kappa range: 0.42–0.85) [37,55,56]. However, agreement improved to very good (kappa = 0.870) when evaluating the entire slide.

This study provides additional evidence to support the existing literature, which also reports a relationship between stromal percentage and cancer metastasis in colorectal cancer. The focus on a well-defined, relatively homogeneous cohort and the long median follow-up of 161 months are major strengths of the study.

However, one of its main limitations is that the number of high-stroma cases is relatively small (16 of 131), which is reflected in the somewhat wide confidence intervals for the hazard ratios (Table 2). For this reason, these results support but do not definitively establish stroma as an independent prognostic factor in stage II microsatellite-stable disease.

Other limitations of our study include not evaluating known molecular prognostic alterations such as RAS, BRAF, HER2, NTRK, or POLE mutations.

Furthermore, we used an older patient cohort (years 2000–2005). The mainstay treatment for this tumor type continues to rely on 5-fluorouracil-based regimens. Attempts at targeted therapies, such as anti-VEGF agents, have not demonstrated significant differences between groups [57] and underscores the urgent need for new targeted treatments through clinical trials.

Future directions of research include conducting prospective studies to better identify patients suitable for clinical trials, evaluating the impact of stromal percentage assessment in preoperative biopsies, and applying computational pathology approaches to infer molecular alterations from surrogate phenotypic features such as stromal percentage.

In summary, we have addressed the proposed objectives: (1) we found an association between stromal percentage and tumor budding, but not with other clinicopathological variables; (2) we demonstrated a markedly poorer prognosis in patients with stroma-rich tumors, with higher recurrence rates; and (3) we observed moderate interobserver agreement for the single-field method and high agreement with the global assessment approach.

## 4. Materials and Methods

### 4.1. Ethical Considerations

This study was conducted in accordance with the principles of the Declaration of Helsinki and was approved by the Ethics Committee of the Complejo Hospitalario Universitario de Toledo (approval code: 585, approval date: 26 October 2020).

### 4.2. Patient Selection

All patients diagnosed with stage II colorectal adenocarcinoma between 2000 and 2005 at our institution (Complejo Hospitalario Universitario de Toledo)were included.

Exclusion criteria were hereditary syndromes, loss of nuclear expression of DNA mismatch repair proteins (as a surrogate marker of microsatellite instability), cases receiving neoadjuvant therapy, or tumors with a neuroendocrine component.

Mismatch repair proteins were analyzed in all cases by immunohistochemistry (MLH1, clone ES05 -*Agilent Dako*-; PMS2, clone EP51 -*Agilent Dako*-; MSH2, clone FE11 -*Agilent Dako*-; and MSH6, clone EP49 -*Agilent Dako*-).

After this, a total of 131 patients from the University Hospital of Toledo were included.

### 4.3. Study Variables

The following variables were collected: sex, age, tumor location, histological type, pT stage, number of isolated lymph nodes, lymphatic invasion, vascular invasion, perineural invasion, tumor budding, histological grade, stromal percentage, surgical margin status, tumor obstruction/perforation, adjuvant therapy, and recurrence.

Age was categorized as ≤70 or >70 years in accordance with previous studies [58].

Lymphatic invasion was differentiated from venous vascular invasion based on wall characteristics, and the use of D240 immunohistochemistry in suspected cases, with positive results in lymphatics and negative results in veins.

The stromal percentage was measured globally on the slide containing the invasive tumor front and classified as high when >50% and low when ≤50% (Figure 1). The entire infiltrating component should be counted, excluding mucin, necrosis, muscle layers, and large vessels [23].

Adjuvant therapy was administered according to the guidelines of the Spanish Society of Medical Oncology and the ESMO at the time of this historical cohort (years 2000–2005).

### 4.4. Statistical Analysis

Statistical analysis was performed using SPSS version 29 (IBM Corp., Armonk, NY, USA).

For the descriptive analysis, absolute frequencies and percentages were used.

Bivariate associations between qualitative variables were evaluated using the chi-square test or Fisher’s exact test for small samples.

The median follow-up time was calculated using the reverse Kaplan–Meier estimator.

Survival analysis for DFS was performed using the Kaplan–Meier method. Comparisons between survival curves were made using the log-rank test. DFS was chosen as the primary endpoint for this study, as it directly reflects the impact of tumor characteristics on disease recurrence and is less affected by comorbidities or other non-cancer-related factors compared with overall survival.

A multivariate Cox regression analysis was performed. Using the simultaneous covariate entry method (not stepwise), all listed variables were included except for the number of retrieved lymph nodes, surgical margin status, and tumor obstruction/perforation, which were not included because none of the cases in this series had fewer than 12 isolated lymph nodes, positive surgical margins, or evidence of tumor obstruction/perforation. Proportional hazards assumptions were assessed using the Schoenfeld test.

For the interobserver agreement analysis, six pathologists independently evaluated a total of 60 cases using both the global stromal measurement method and the single-field method. Agreement was assessed using Fleiss’ Kappa index, interpreted as follows: <0.20 = poor agreement, 0.21–0.40 = fair, 0.41–0.60 = moderate, 0.61–0.80 = good, and 0.81–1.00 = very good [59].

A two-tailed *p*-value < 0.05 was considered statistically significant.

## 5. Conclusions

Tumor stroma may represent a particularly relevant prognostic factor in stage II colorectal adenocarcinomas. Our independent retrospective cohort study, focusing on microsatellite-stable cases, supports this hypothesis. We observed worse disease-free survival in tumors with a high stromal percentage (>50%) upon a global assessment on H&E at the invasive front, which is a simple and highly reproducible method, showing very good interobserver agreement. The current clinical assessment suggests that its measurement may help identify patients who could benefit from future drugs while avoiding the toxicities of current, minimally effective treatments in this type of tumor.

## Figures and Tables

**Table 1:** Association between stromal percentage and other relevant prognostic factors.

Characteristics	Low Stroma (n, %)	High Stroma (n, %)	*p*-Value
Cases (n = 131)	115 (87.8)	16 (12.2)	-
***Sex***			
Female	37 (32.2)	5 (31.2)	0.941
Male	78 (67.8)	11 (68.8)	
***Age***			
≤70 Years	42 (36.5)	3 (18.7)	0.260 *
>70 Years	73 (63.5)	13 (81.3)	
***Tumor Location***			
Right Colon	34 (29.6)	5 (31.2)	0.890
Left Colon–Rectum	81 (70.4)	11 (68.8)	
***Histology***			
NOS	100 (87)	14 (87.5)	1.000 *
Others	15 (13)	2 (12.5)	
***pT Stage***			
pT3	36 (31.3)	6 (37.5)	0.619
pT4 (pT4a–pT4b)	79 (68.7)	10 (62.5)	
***Lymphatic Invasion***			
Absent	110 (95.7)	15 (93.8)	0.550 *
Present	5 (4.3)	1 (6.2)	
***Vascular Invasion***			
Absent	89 (77.4)	12 (75)	0.761 *
Present	26 (22.6)	4 (25)	
***Perineural Invasion***			
Absent	103 (89.6)	15 (93.8)	1.000 *
Present	12 (10.4)	1 (6.2)	
***Tumor Budding***			
Low–Intermediate (0–9)	114 (99.1)	13 (81.3)	0.006 *
High (≥10)	1 (0.9)	3 (18.7)	
***Histologic Grade***			
Low (≥50% Glandular)	105 (91.3)	15 (93.8)	1.000 *
High (<50% Glandular)	10 (8.7)	1 (6.2)	
***Adjuvant Therapy***			
No	32 (36.8)	5 (50)	0.415
Yes	55 (63.2)	5 (50)	
***Recurrence***			
No	82 (71.3)	6 (37.5)	0.007
Yes	33 (28.7)	10 (62.5)	

* Fisher’s exact test (if n < 5).

**Table 2:** Cox regression data for different prognostic factors.

Variable	Hazard Ratio	95% CI	*p*-Value
***Sex***			
Female	Ref.	-	-
Male	1.168	0.498–2.741	0.722
***Age***			
≤70 Years	Ref.	-	-
>70 Years	1.136	0.469–2.749	0.778
***Tumor Location***			
Right Colon	Ref.	-	-
Left Colon–Rectum	1.627	0.623–4.248	0.320
***Histology***			
NOS	Ref.	-	-
Others	0.947	0.296–3.025	0.926
***pT Stage***			
pT3	Ref.	-	-
pT4 (pT4a–pT4b)	1.217	0.514–2.885	0.655
***Lymphatic Invasion***			
Absent	Ref.	-	-
Present	16.513	3.506–77.769	<0.001
***Vascular Invasion***			
Absent	Ref.	-	-
Present	0.260	0.055–1.234	0.090
***Perineural Invasion***			
Absent	Ref.	-	-
Present	2.060	0.502–8.447	0.316
***Tumor Budding***			
Low–Intermediate (0–9)	Ref.	-	-
High (≥10)	1.878	0.208–16.965	0.575
***Histologic Grade***			
Low (≥50% glandular)	Ref.	-	-
High (<50% glandular)	2.164	0.669–6.999	0.198
***Tumor Stroma***			
Low (≤50%)	Ref.	-	-
High (>50%)	4.366	1.516–12.576	0.006
***Adjuvant Therapy***			
No	Ref.	-	-
Yes	0.814	0.312–2.127	0.675

Ref.: Reference value.

## Data Availability

The original contributions presented in this study are included in the article. Further inquiries can be directed to the corresponding author.
